# Developmental aspects of cortical excitability and inhibition in depressed and healthy youth: an exploratory study

**DOI:** 10.3389/fnhum.2014.00669

**Published:** 2014-09-02

**Authors:** Paul E. Croarkin, Paul A. Nakonezny, Charles P. Lewis, Michael J. Zaccariello, John E. Huxsahl, Mustafa M. Husain, Betsy D. Kennard, Graham J. Emslie, Zafiris J. Daskalakis

**Affiliations:** ^1^Division of Child and Adolescent Psychiatry, Department of Psychiatry and Psychology, Mayo ClinicRochester, MN, USA; ^2^Division of Biostatistics, Department of Clinical Sciences, UT Southwestern Medical CenterDallas, TX, USA; ^3^Department of Psychiatry, UT Southwestern Medical CenterDallas, TX, USA; ^4^Department of Psychiatry and Behavioral Sciences, Duke University School of MedicineDurham, NC, USA; ^5^Department of Psychiatry, Centre for Addiction and Mental Health, University of TorontoToronto, ON, Canada

**Keywords:** adolescents, depression, neurodevelopment, CSP, ICF, SICI, LICI, TMS

## Abstract

**Objectives:** The objective of this *post-hoc* exploratory analysis was to examine the relationship between age and measures of cortical excitability and inhibition.

**Methods**: Forty-six participants (24 with major depressive disorder and 22 healthy controls) completed MT, SICI, ICF, and CSP testing in a cross-sectional protocol. Of these 46 participants, 33 completed LICI testing. Multiple linear robust regression and Spearman partial correlation coefficient were used to examine the relationship between age and the TMS measures.

**Results**: In the overall sample of 46 participants, age had a significant negative relationship with motor threshold (MT) in both the right (*r*_*s*_ = −0.49, adjusted *p* = 0.007; β = −0.08, adjusted *p* = 0.001) and left (*r*_*s*_ = −0.42, adjusted *p* = 0.029; β = −0.05, adjusted *p* = 0.004) hemispheres. This significant negative relationship of age with MT was also observed in the sample of depressed youth in both the right (*r*_*s*_ = −0.70, adjusted *p* = 0.002; β = −0.09, adjusted *p* = 0.001) and left (*r*_*s*_ = −0.54, adjusted *p* = 0.034; β = −0.05, adjusted *p* = 0.017) hemispheres, but not in healthy controls. In the sample of the 33 participants who completed LICI testing, age had a significant negative relationship with LICI (200 ms interval) in both the right (*r*_*s*_ = −0.48, adjusted *p* = 0.05; β = −0.24, adjusted *p* = 0.007) and left (*r*_*s*_ = −0.64, adjusted *p* = 0.002; β = −0.23, adjusted *p* = 0.001) hemispheres. This negative relationship between age and LICI (200 ms interval) was also observed in depressed youth in both the right (*r*_*s*_ = −0.76, adjusted *p* = 0.034; β = −0.35, adjusted *p* = 0.004) and left (*r*_*s*_ = −0.92, adjusted *p* = 0.002; β = −0.25, adjusted *p* = 0.001) hemispheres.

**Conclusion**: These findings suggest that younger children have higher MTs. This is more pronounced in depressed youth than healthy controls. LICI inhibition may also increase with age in youth.

## Introduction

Transcranial magnetic stimulation (TMS) measures of cortical excitability and inhibition have shown initial promise as biomarkers and for the study of neurophysiology in youth with attention deficit hyperactivity disorder (Gilbert et al., 2011), neurodevelopmental disorders (Garvey et al., 2001, 2003; Garvey and Gilbert, 2004; Enticott et al., 2013; Oberman et al., 2013) and mood disorders (Croarkin et al., 2013, 2014). These TMS paradigms involve quantifying the effects of brief TMS pulses on the motor cortex through the measurement of a motor evoked potential (MEP) with surface electromyography (EMG) applied to hand muscles such as the abductor pollicis brevis (APB) (Levin et al., 2014). The amplitude of a MEP reflects the excitatory/inhibitory balance of cortical pyramidal cells. Based on prior work, these measures have good reliability and validity (Farzan et al., 2010). Notably, many of these measures are indirect indices gamma-aminobutyric acid (GABA) and glutamate receptor mediated neurotransmission (Radhu et al., 2013).

Two measures of cortical excitability are the motor threshold (MT) which is a single pulse TMS measure and intracortical facilitation (ICF) which is a paired-pulse TMS measure. Prior human pharmacologic studies indicate that the MT is at least partially dependent on voltage-gated sodium channels while ICF is an indirect measure of glutamatergic N-methyl-D-aspartate (NMDA) mediated neurotransmission (Ziemann et al., 1996a,b, 1998). The MT is operationally defined as the stimulus intensity which produces a reliable MEP with a stimulation intensity of at least 50 microvolts in 5 out of 10 trials. During ICF measures, a subthreshold condition stimulation (set to 80% of resting MT) precedes a suprathreshold test stimulation with interstimulus intervals of 10–20 ms. The conditioned MEP is compared to the MEP produced by a suprathreshold test stimulus to examine the degree of change in amplitude. Higher ratios reflect increased cortical facilitation. Measures of cortical inhibition include short-interval intracortical inhibition (SICI) (Kujirai et al., 1993), long-interval cortical inhibition (LICI) (Daskalakis et al., 2008; Farzan et al., 2010), and the cortical silent period (CSP) (Garvey and Mall, 2008). Prior research suggests that SICI is a measure of GABA_A_ receptor mediated neurotransmission (Kujirai et al., 1993; Paulus et al., 2008), while LICI and CSP index GABA_B_ receptor mediated neurotransmission (Connors et al., 1988; Kujirai et al., 1993; McDonnell et al., 2006). For SICI measures, a subthreshold conditioning stimulation (set to 80% of resting MT) precedes a suprathreshold test stimulation with interstimulus intervals of 1–5 ms. The conditioned MEP is compared to the MEP produced by a suprathreshold test stimulus to examine the degree of change in amplitude. Lower SICI ratios reflect increased cortical inhibition. During LICI measurements, suprathreshold conditioning and suprathreshold test stimulations are applied to the motor cortex with 50–200 ms interstimulus intervals (Kujirai et al., 1993; Garvey and Mall, 2008). The conditioned MEP is compared to the MEP produced by a suprathreshold test stimulus to examine the degree of change in amplitude. Lower LICI ratios reflect increased cortical inhibition. During CSP testing the participant contracts the muscle of interest submaximally while a suprathreshold, single-pulse stimulation is administered to the motor cortex. The resultant period of EMG silence corresponds to the amount of cortical inhibition (Farzan et al., 2013). Contralateral silent period measures are typically collected in psychiatric research, although it is possible to collect ipsilateral silent period data as well (Garvey and Gilbert, 2004). Both CSP measures reflect the function of cortical inhibitory interneurons and the corpus callosum (Müller et al., 1997; Garvey et al., 2003).

These neurophysiologic indices have potential for classifying psychiatric illnesses and guiding pharmacologic treatments (Ziemann, 2004; Paulus et al., 2008). Research regarding the developmental courses of cortical excitability and inhibition measures in adulthood is emerging (McGinley et al., 2010; Fling and Seidler, 2012; Heise et al., 2013; Levin et al., 2014; Liguz-Lecznar et al., 2014), but remarkably little is known about the early trajectory of each measure in health and disease (Moll et al., 1999; Mall et al., 2004; Garvey, 2008). Although there is a paucity of research, it is generally accepted that MT measures are higher in children than in adults (Garvey and Gilbert, 2004; Garvey, 2008). At some point in adolescence, MT values fall to adult levels (Garvey and Gilbert, 2004). Some TMS studies suggest that the CSP durations increase with age while other studies others do not (Moll et al., 1999; Garvey et al., 2003). Research regarding early age-related differences in SICI and ICF is also inconclusive (Moll et al., 1999; Garvey et al., 2003; Mall et al., 2004; Gilbert et al., 2011). Further, there are few published reports examining LICI measures in children and adolescents (Croarkin et al., 2014). An enhanced understanding of developmental changes in these neurophysiologic measures would have great utility. This knowledge would advance understanding of neurodevelopment, the ontogeny of GABA and glutamate neurotransmission, and inform neurophysiologic study protocols (Paulus et al., 2008; Radhu et al., 2013). The aim of this exploratory study was to examine the current suppositions regarding developmental changes in motor cortex excitability and inhibition in early life. In particular, we examined the association of age with cortical inhibition (SICI, LICI, and CSP) and excitability (MT, ICF).

## Methods

### Study design and overview

This was a cross-sectional study of depressed youth and healthy controls. All participants had a clinical evaluation and TMS testing. Cortical inhibition (SICI, CSP) and excitability (MT and ICF) were collected during a single session. A subgroup of this cohort completed LICI testing during the same testing session. Study design details have been previously published (Croarkin et al., 2013, 2014). All study procedures were approved by the local institutional review board prior to the enrollment of subjects. Below is a brief description of relevant design aspects of this study.

### Study participants

This study involved the 46 male and female, children and adolescents, aged 9–17 years from our parent study (Croarkin et al., 2013, 2014). In particular, 24 participants had major depressive disorder (MDD) and 22 participants were healthy controls. Of the 46 youth in our parent study (Croarkin et al., 2013, 2014), 33 of these participants (14 with MDD and 19 healthy controls) completed the LICI testing protocol. This sample was recruited from a pediatric mood disorders clinic and local advertising. After obtaining written assent from youth and informed consent from legal guardians, participants, and families were evaluated by a board-certified child and adolescent psychiatrist (P.E.C.). This included a clinical history, psychiatric history, medical history, mental status exam, neurological exam, physical exam, urine pregnancy test for females who had reached menarche, and a semi-structured interview with Schedule for Affective Disorders and Schizophrenia, Present and Lifetime Version (K-SADS-PL) (Kaufman et al., 1997). Depression severity was assessed with the Children's Depression Rating Scale, Revised (CDRS-R) (Poznanski et al., 1983). The Oldfield's Edinburgh Handedness Inventory (Oldfield, 1971) was completed to confirm handedness. The TMS Adult Safety Screen (Keel et al., 2001) was used to confirm safety for single- and paired-pulse TMS testing. Depressed patients and healthy controls were not taking psychotropic medications or receiving psychotherapy prior to the TMS testing session.

A personal or family history of seizure disorders was exclusionary. A prior history of neurosurgery was exclusionary. Exclusionary psychiatric disorders in depressed participants included autism spectrum disorders, bipolar disorder, conduct disorder, eating disorders, mental retardation, obsessive-compulsive disorder, posttraumatic stress disorder, schizophrenia, substance use disorders, and tic disorders. Screening for mental retardation involved a detailed review of records and questions regarding academic performance. If below-average cognitive ability was considered, formal intellectual screening was completed with the Kaufman Brief Intelligence Test-2 (Kaufman and Kaufman, 2004). This encompassed verbal knowledge, matrices, and riddles. Youth with an estimated IQ less than 80 were not enrolled. Healthy control participants were in excellent health and did not meet present or past diagnostic criteria for any psychiatric illness based on the evaluation and the K-SADS-PL interview.

### Procedures and measures

#### TMS testing

Cortical excitability and inhibition TMS testing was performed as described in prior literature (Kujirai et al., 1993; Ziemann, 2004; Paulus et al., 2008). Participants were seated and wore a swim cap during the procedures. Participants and study team wore earplugs during the testing. Stimulations were delivered with a Magstim 200 magnetic simulator (Magstim Co Ltd.) with a figure-of-eight coil (each loop is 70 mm in diameter). MEP data were collected with surface EMG recordings of the APB muscle. Audio feedback was examined to assess muscle relaxation of the participant. Single and paired-pulse TMS was delivered to the hand area of the contralateral motor cortex with the coil placed tangentially on the scalp at a 45° from the midline. The motor cortex testing site was ascertained after moving the coil in 1-cm increments to locate the site producing maximal MEP. The location was marked for reliable testing throughout the session. The resting MT was established as the stimulation intensity producing a MEP greater than 50 μV in 5 of 10 TMS pulses with a relaxed muscle. The suprathreshold test stimulus was obtained but adjusting stimulation intensity to produce a mean MEP 0.5–1.5 mV peak-to-peak in amplitude in the dominant hand muscle. The conditioning stimulus was set to 80% of the participants resting MT. For SICI and ICF measures, the conditioning and test stimuli were delivered to the test site at interstimulus intervals of 2, 4, 10, 15, 20 ms in a random, counterbalanced fashion. Each interstimulus interval was tested 12 times and results were averaged. The left hemisphere was stimulated first in all subjects. For LICI testing, test stimulus intensity was adjusted to reliably produce MEP 0.5–1.5 mV peak-to-peak in amplitude in the dominant hand muscle. For LICI testing the conditioning and test stimuli were both delivered at this suprathreshold intensity with interstimulus intervals of 100, 150, and 200 ms in a random, counterbalanced fashion. Each interstimulus interval was tested 10 times and results were averaged. The MEP data from these paired-pulse TMS measures was expressed as the percentage of the mean MEP elicited with the test (unconditioned pulse). For CSP testing, the participant submaximally (20%) contracted the APB with contralateral single-pulse stimulations at 140% of the participants resting MT. Ten trials were performed and averaged to determine the durations of the silent period. The TMS testing was performed bilaterally for this study providing data from both hemispheres.

#### Dependent variables

The outcomes were TMS measures of cortical inhibition (SICI, LICI, CSP) and excitability (MT, ICF). The TMS measures were log transformed to obtain a more normal distribution (because of skewness).

#### Independent variable and covariates

The primary independent variable was patient age in years. Sex and CDRS-R total score were included as covariates in the models to bolster precision in the evaluation of the relationship between age and each measure of cortical inhibitory or excitatory functioning. Some (Cuypers et al., 2014) but not all (Wasserman, 2002) studies suggest that sex may contribute to variation in TMS MEP measures. Although, it is not well understood, depression severity (CDRS-R) may also impact TMS MEP measures (Croarkin et al., 2013).

### Statistical analysis

This was an exploratory study with the aim of investigating the impact of age on measures of cortical inhibition and excitability in youth. Analyses were performed on the entire sample and then on subgroups of healthy and depressed youth. The rationale for this approach was to maximize limited data and with the realization that the impact of depression or depression severity on these neurophysiological measures is not definitively known. Demographic and clinical characteristics for the overall sample of patients and for depressed youth and healthy controls were described using the sample mean and standard deviation for continuous variables and the frequency and percentage for categorical variables. Multiple linear robust regression (with MM estimation) and the Spearman partial correlation coefficient (*r*_*s*_) were used to examine the relationship between age and TMS measures of cortical inhibition (SICI, LICI, CSP) and excitability (MT, ICF) in the overall sample, while adjusting for sex and CDRS-R total score, and then separately in depressed youth and healthy controls, while adjusting for sex. The estimated slope from the regression model indicates the mean change in each TMS measure per one-year increase in age, while the sample correlation coefficient indicates not only direction, but also strength of the linear relationship between age and each TMS measure. The Spearman partial correlation coefficient can also be interpreted as the effect size estimator in evaluating the magnitude of the relationship between age and each TMS measure.

Regression coefficients found to have statistical significance for each of the two groups in the abovementioned regression analysis, were then tested in subsequent (*post-hoc*) regression models (similar to that described above) by evaluating the interaction effect of group (Healthy Controls vs. MDD Patients) with age on the TMS measure. Comparing the regression parameters (slopes) between groups was used to assess if the mean change in the TMS measure per one-year increase in age was different between healthy controls and MDD patients.

All statistical analyses were performed using SAS software, version 9.3 (SAS Institute, Inc., Cary, NC, USA). The level of significance for all tests was set at α = 0.05 (two-tailed). We implemented the False Discovery Rate procedure (Benjamini and Hochberg, 1995) to control false-positives over the sets of multiple tests associated with the correlation coefficient and regression coefficient for the groups of participants (e.g., overall sample, depressed youth, and healthy controls).

## Results

### Participant characteristics

Twenty-four, medication-naïve participants (14 female) with MDD completed MT, SICI, ICF, and CSP testing. Demographics of the sample are also characterized our prior publication. In comparing participants with MDD and healthy controls, there were no statistically significant differences in age, sex, or handedness (Croarkin et al., 2013). These participants were 9–17 years of age, with a mean [SD] age of 13.8 [2.1] years. The mean [SD] CDRS-R total score of the MDD youth was 58.9 [8.5] and the mean [SD] episode duration was 10.9 [9.7] months. Eight participants with MDD had comorbid anxiety disorders, four had comorbid attention-deficit/hyperactivity disorder, one had comorbid oppositional defiant disorder, and one had comorbid Type I diabetes mellitus. Two MDD participants were left handed. Twenty-two healthy controls (11 female) completed MT, SICI, ICF, and CSP testing. These participants were 9–17 years of age, with a mean [SD] age of 13.7 [2.2] years. The mean [SD] CDRS-R total score of the healthy controls was 19.6 [1.6]. Two healthy controls were left handed. Of the 46 adolescents in the current study, 23 (50%) were Caucasian, 13 (28.2%) were African American, 4 (8.7%) were Hispanic, and 13 (28.2%) had a family history of mood disorder. Family history of mood disorder occurred in 13 of the 24 depressed adolescents (54.2%) and in 0 of the 22 healthy controls. Fourteen patients with MDD (8 female), with a mean [SD] age of 14.0 [2.1] years, completed LICI testing. The mean [SD] CDRS-R total score of these 14 MDD LICI youth was 59.0 [9.6]. Nineteen healthy controls (11 female) with a mean [SD] age of 13.9 [2.2] years completed LICI testing. The mean [SD] CDRS-R total score of these 19 healthy control LICI youth was 19.6 [1.7]. Two of these healthy controls who completed LICI testing were left handed.

### Age and TMS measures of cortical inhibition and excitability

**Figure 1:**
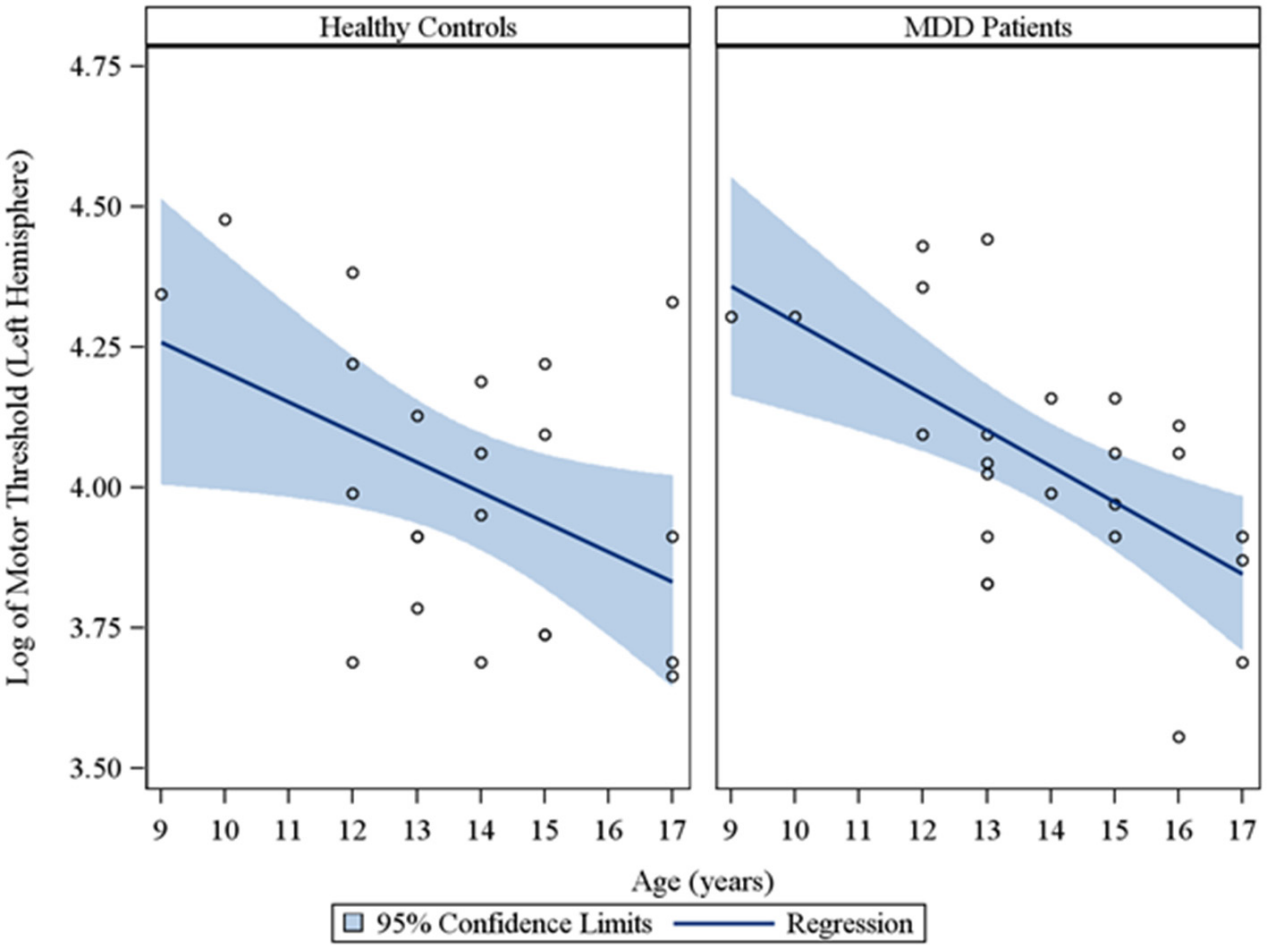
**Scatterplots of the log transformed MT values (left hemisphere) against age, with a fitted regression line and 95% confidence limits, by healthy controls and MDD patients**. Note: The interaction of group with age indicated that the slope of the regression of motor threshold on age was similar for both healthy controls and MDD patients in the left hemisphere (*p* = 0.61).

**Figure 2:**
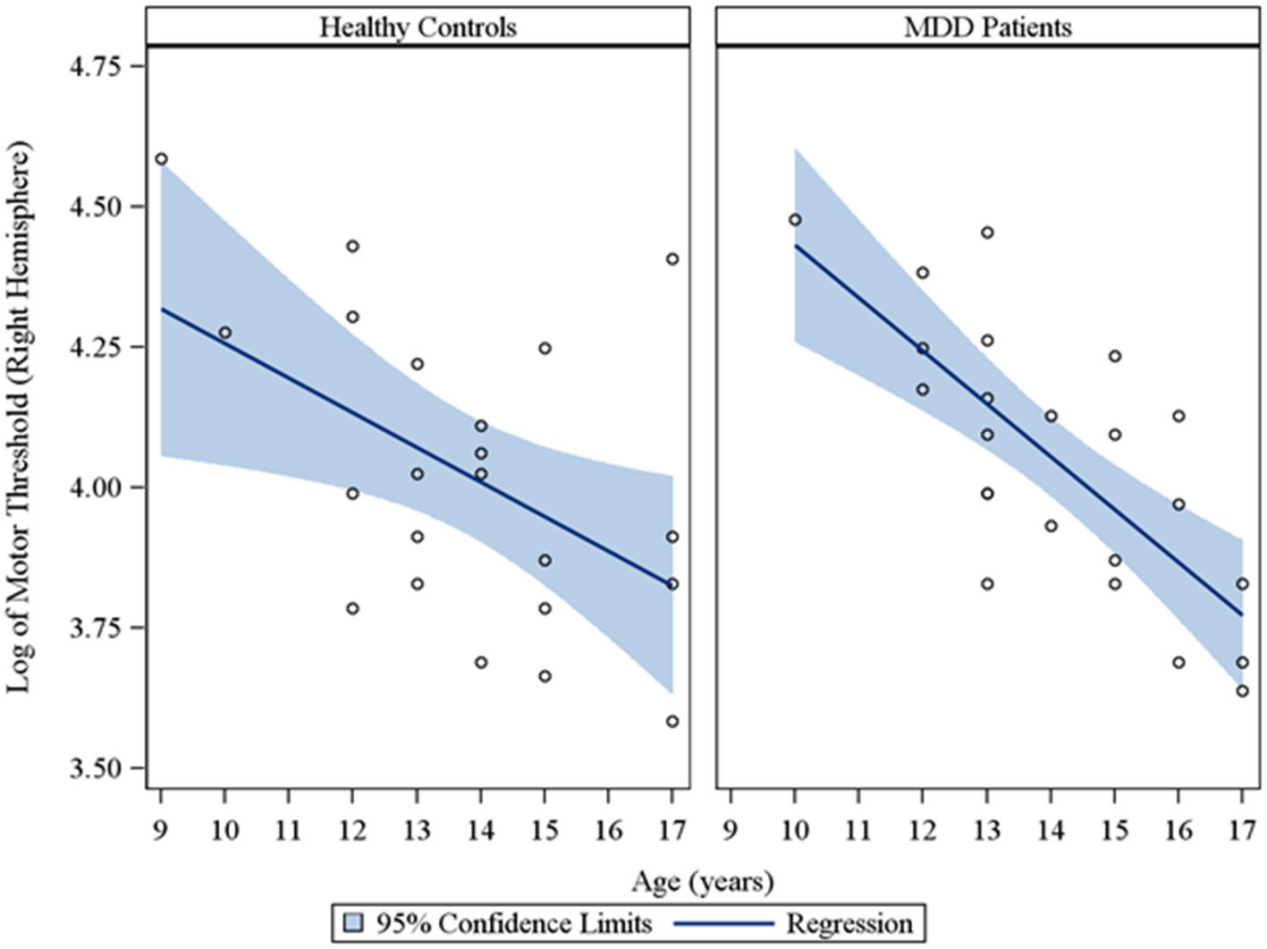
**Scatterplots of the log transformed MT values (right hemisphere) against age, with a fitted regression line and 95% confidence limits, by healthy controls and MDD patients**. Note: The interaction of group with age indicated that the slope of the regression of motor threshold on age was similar for both healthy controls and MDD patients in the right hemisphere (*p* = 0.28).

In the overall sample of 46 youth, the Spearman partial correlations and the multiple linear robust regression, while adjusting for sex and CDRS-R total, revealed a significant negative linear relationship between age and MT in both the right (*r*_*s*_ = −0.49, raw *p* = 0.0007, FDR-adjusted *p* = 0.007; β = −0.08, 95% *CI* = −0.12 to −0.05, raw *p* = 0.0001, FDR-adjusted *p* = 0.001) and left (*r*_*s*_ = −0.42, raw *p* = 0.004, FDR-adjusted *p* = 0.029; β = −0.05, 95% *CI* = −0.09 to −0.02, raw *p* = 0.0007, FDR-adjusted *p* = 0.004) hemispheres. This significant negative relationship of age with MT, while adjusting for sex, was also observed in the sample of depressed youth in both the right (*r*_*s*_ = −0.70, raw *p* = 0.0002, FDR-adjusted *p* = 0.002; β = −0.09, 95% *CI* = −0.13 to −0.05, raw *p* = 0.0001, FDR-adjusted *p* = 0.001) and left (*r*_*s*_ = −0.54, raw *p* = 0.007, FDR-adjusted *p* = 0.034; β = −0.05, 95% *CI* = −0.09 to −0.02, raw *p* = 0.003, FDR-adjusted *p* = 0.017) hemispheres, but not in healthy controls. Note that lower MT values reflect increased excitability. We also present scatterplots of the log transformed MT values (left and right hemispheres) against age, with a fitted regression line and 95% confidence limits, by depressed youth and healthy controls (Figures 1, 2). We note that the *post-hoc* results of the interaction of group with age indicated that the slope of the regression of MT on age was similar for both healthy controls and MDD patients in both the left (*p* = 0.61; Figure 1) and right (*p* = 0.28; Figure 2) hemispheres.

**Figure 3:**
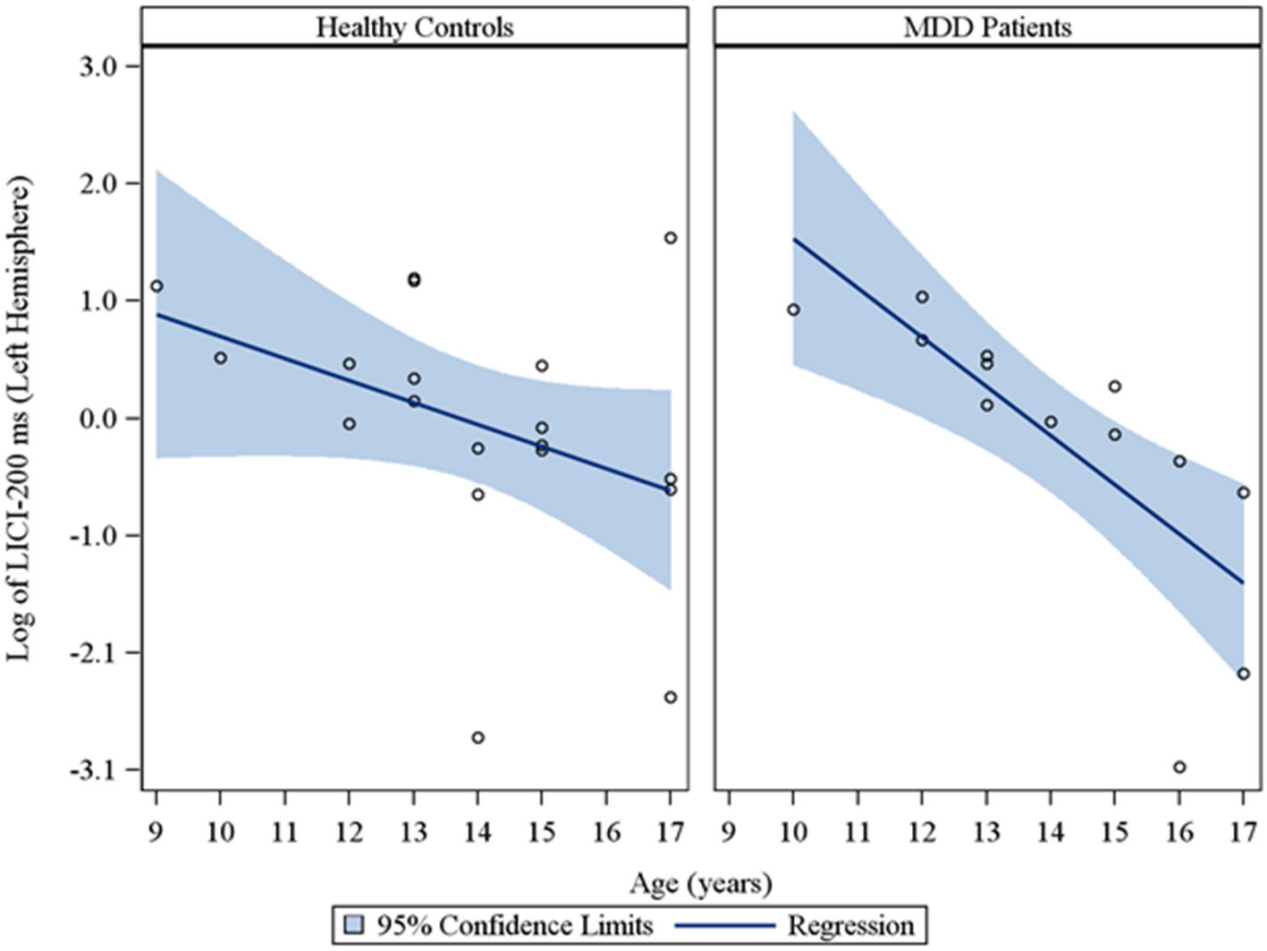
**Scatterplots of the log transformed LICI (200 ms interval) values (left hemisphere) against age, with a fitted regression line and 95% confidence limits, by healthy controls and MDD patients**. Note: The interaction of group with age indicated that the slope of the regression of LICI-200 ms on age was similar for both healthy controls and MDD patients in the left hemisphere (*p* = 0.14).

**Figure 4:**
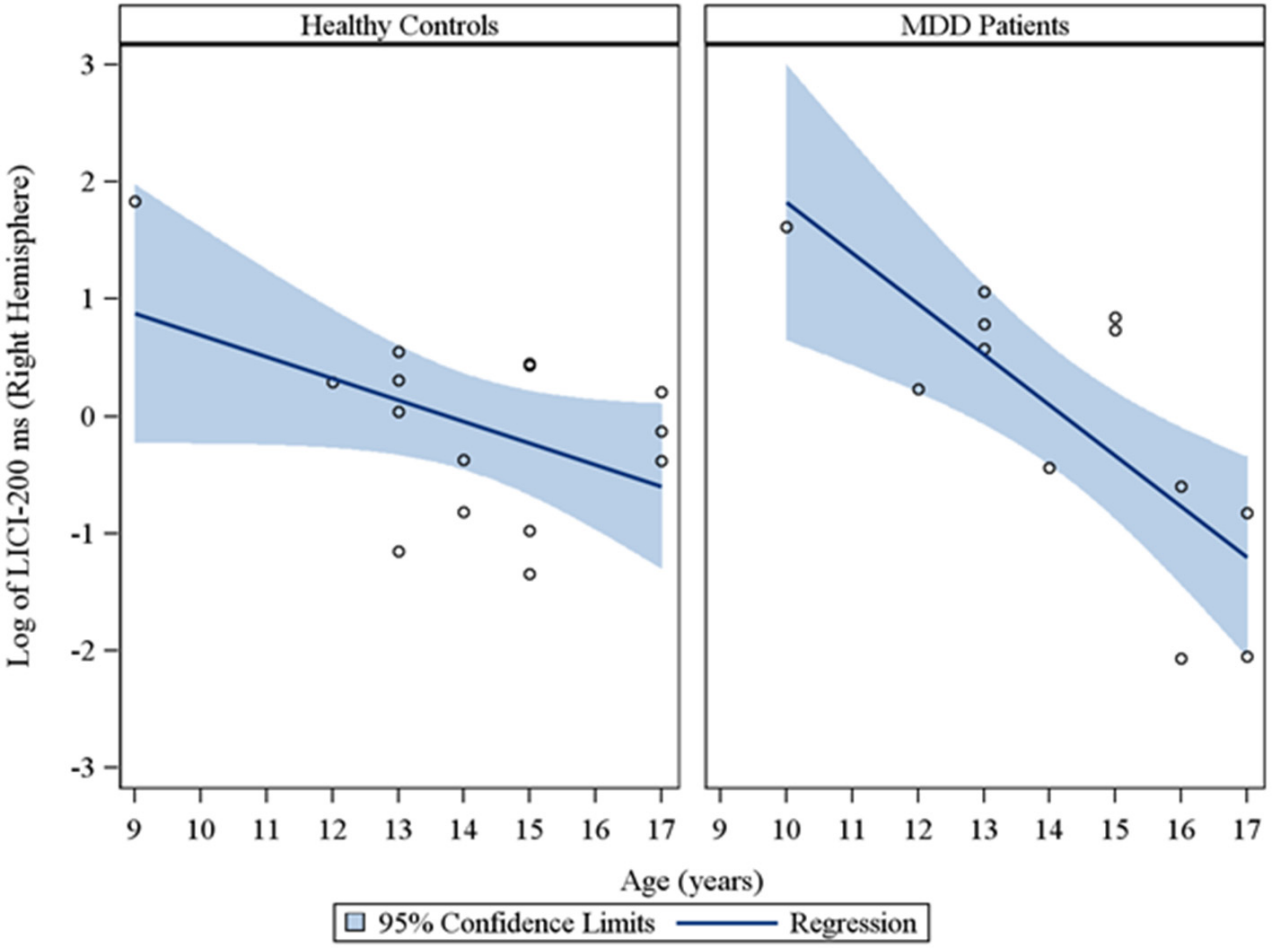
**Scatterplots of the log transformed LICI (200 ms interval) values (right hemisphere) against age, with a fitted regression line and 95% confidence limits, by healthy controls and MDD patients**. Note: The interaction of group with age indicated that the slope of the regression of LICI-200 ms on age was significantly different for healthy controls vs. MDD patients in the right hemisphere (*p* = 0.049).

Moreover, in the sample of the 33 participants who completed LICI testing, while adjusting for sex and CDRS-R total, age had a significant negative relationship with LICI (200 ms interval) in both the right (*r*_*s*_ = −0.48, raw *p* = 0.01, FDR-adjusted *p* = 0.056; β = −0.24, 95% *CI* = −0.39 to −0.09, raw *p* = 0.001, FDR-adjusted *p* = 0.007) and left (*r*_*s*_ = −0.64, raw *p* = 0.0001, FDR-adjusted *p* = 0.002; β = −0.23, 95% *CI* = −0.32 to −0.14, raw *p* = 0.0001, FDR-adjusted *p* = 0.001) hemispheres. This negative relationship between age and LICI (200 ms interval), while adjusting for sex, was also observed in depressed participants (Supplementary Table 5) in both the right (*r*_*s*_ = −0.76, raw *p* = 0.006, FDR-adjusted *p* = 0.034; β = −0.35, 95% *CI* = −0.55 to −0.15, raw *p* = 0.0007, FDR-adjusted *p* = 0.004) and left (*r*_*s*_ = −0.92, raw *p* = 0.0001, FDR-adjusted *p* = 0.002; β = −0.25, 95% *CI* = −0.34 to −0.16, raw *p* = 0.0001, FDR-adjusted *p* = 0.001) hemispheres and in healthy controls (Supplementary Table 6), but only in the left hemisphere (*r*_*s*_ = −0.45, raw *p* = 0.05, FDR-adjusted *p* = 0.877; β = −0.19, 95% *CI* = −0.36 to −0.02, raw *p* = 0.02, FDR-adjusted *p* = 0.478). Note that lower LICI values reflect increased inhibition. Scatterplots of the log transformed LICI (200 ms interval) values (left and right hemispheres) against age, with a fitted regression line and 95% confidence limits, by depressed youth and healthy controls are presented in Figures 3, 4. We note that the *post-hoc* results of the interaction of group with age indicated that the slope of the regression of LICI-200 ms on age was similar for both healthy controls and MDD patients in the left hemisphere (*p* = 0.14; Figure 3), but significantly different in the right hemisphere (*p* = 0.049; Figure 4). A difference in slopes here (in the right hemisphere) can be interpreted as differences in the mean change in LICI-200 ms per 1-year increase in age between healthy controls and MDD patients. There were no significant relationships of age with SICI, CSP, or ICF. The correlation and regression results are presented in Tables 1–6 which are available in the Supplementary Material. Note that nontransformed data of these TMS measures from this cohort have been published previously (Croarkin et al., 2013).

## Discussion

This exploratory study builds on prior work examining the early developmental course of cortical inhibition and excitability measures. It is unique in examining these electrophysiological findings in the context of MDD. As expected, the findings suggest that younger children have high MTs which decrease with age. Of note, this was statistically significant in the depressed participant group, but not in the healthy control group. The current, developmental MT findings are congruent with prior work (Eyre et al., 1991). Garvey et al. demonstrated that bilateral MT measurements decreased with age and in three subjects with a mean age of 7.6 years (*SD* 0.5, range 7–7.8) the MT was unobtainable with maximum output of the stimulator (Garvey et al., 2003). Gilbert and colleagues also reported statistically significant, negative correlations between age and MT measures from the left hemisphere in 98 (49 with attention-deficit/hyperactivity disorder and 49 healthy control subjects) child participants who were 8–12 years of age (Gilbert et al., 2011). Moll and colleagues reported similar findings in 40 healthy control subjects who were 8–16 years of age (Moll et al., 1999). The present study is the first to note this pattern in a group of depressed and healthy control subject. This finding warrants further consideration and study. Perhaps, healthy control youth subjects reach an adult MT earlier, while depressed youth have a more delayed course. It is possible that differences in scalp to cortex distances could play a role in these age differences. Recent work demonstrates that brain-scalp differences are lower in young children and increase throughout childhood (Beauchamp et al., 2011). Based on this anatomical finding alone, it might be inferred that lower TMS current would be sufficient to induce motor activity in younger children and that younger children would have lower MTs. As this does not appear to be the case, age related differences are more likely due to neurodevelopmental changes.

The present CSP, SICI, and ICF findings are inconclusive. Similarly, findings from two prior studies also found no statistically significant relationships between age and CSP measures (Garvey et al., 2003; Gilbert et al., 2011). However, Moll and colleagues reported a correlation with age and CSP in healthy controls which suggests that cortical inhibition and GABA_B_ mediated neurotransmission is potentiated with age (Moll et al., 1999). It is often noted that CSP measures have large interindividual variability in children and adolescents (Garvey and Mall, 2008). Further, hemispheric differences are also apparent (Garvey, 2008; Garvey and Mall, 2008; Croarkin et al., 2013). In general, our findings and two prior studies suggest that there are no SICI or ICF age dependent relationships in youth (Garvey et al., 2003; Gilbert et al., 2011). However, Mall and colleagues examined SICI in 50 healthy participants aged 6–34 years. In this case, children and adolescents had less SICI as compared to adults, which suggests that cortical inhibition and GABA_A_ mediated neurotransmission develops throughout childhood (Mall et al., 2004). This effort did involve stimulation with a round rather than a figure-of-eight coil but otherwise its methodology was congruent with negative studies.

The current findings suggest that LICI inhibition increases with age youth. In particular, LICI (200 ms interval) had a statistically significant negative relationship with age in depressed youth, but not in healthy control participants. It is important to acknowledge the lack of a relationship may be attributable to low power, given the high degree of variability in the measure in general. Lower LICI ratios signify greater inhibition and increased GABA_B_ mediated neurotransmission (Connors et al., 1988; McDonnell et al., 2006; Daskalakis et al., 2008). Prior work postulates that deficits in GABA_A_ and GABA_B_ mediated neurotransmission play a role in the pathophysiology of MDD (Bajbouj et al., 2006; Levinson et al., 2010; Croarkin et al., 2011). However, GABA_B_ receptors are widely distributed throughout the brain presynaptically and postsynaptically with complex and diverse functioning (Benarroch, 2012). In some instances, facilitated GABA activity likely contributes to neuropsychiatric disease mechanisms. Heightened thalamic postsynaptic GABA_B_ activity is thought to play a central role in the pathophysiology of petit mal seizures (Blumenfeld and McCormick, 2000; McCormick and Contreras, 2001). Other work suggests that that aberrant interplay between GABA and NMDA receptors is necessary for the genesis of hippocampal seizures (Bradford, 1995; Katsumori et al., 1998). Enhanced GABA_B_ development in childhood could represent a vulnerability to or a consequence of mood disorders. Our findings are unique in that there are no other known published reports examining potential relationships of age with LICI measures in child and adolescent samples.

Longitudinal studies of cortical inhibition and excitability during childhood are warranted. The GABAergic and glutamatergic neurotransmitter systems have a complex and poorly understood maturation with which widely influences central nervous system development and functioning. For example, in prenatal and early infantile life GABA serves as an excitatory neurotransmitter and this is mediated primarily by the structure and fuction of GABA receptors (Ben-Ari et al., 1994; Leinekugel et al., 1999; Rakhade and Jensen, 2009). It is thought that density of GABA_A_ receptors decreases from childhood to adulthood (Chugani et al., 2001) and that the subunit composition of GABA_A_ receptors varies substantially during development (Duncan et al., 2010). Lower cortical inhibition early in life may reflect increased plasticity and optimum conditions for learning and development (Mall et al., 2004). Safe, noninvasive, *in vivo* measures afforded by TMS offer an important tool to probe the development of these neurotransmitter systems (Dayan et al., 2013).

## Limitations

These discussion points and findings must be placed in the context of the limitations of the study. This was a *post-hoc* exploratory study, which was based on a small, age-restricted, sample. The parent study (Croarkin et al., 2013, 2014) from which the current findings are based was not designed to specifically address the question of age effects on TMS measures of cortical inhibition and excitability. Like previous studies that examined the effects of age on TMS measures, the current study design was cross-sectional. A longitudinal study would provide more insight into the pattern of findings regarding the impact of development over the lifespan. It is also unknown if alterations in cortical inhibition and excitability measures are deficiencies related to the burden of psychiatric illnesses or markers of risk. Moreover, a cross-sectional approach is limited as it has been demonstrated that TMS measures have wide inter-individual variability (Kiers et al., 1993; Cuypers et al., 2014). The present findings may relate to the ontogeny of GABA and glutamate neurotransmission or could be a result of developmental changes in anatomy, such as skull bone shape and thickness.

## Conclusions

These findings provide additional information regarding the potential impact of age on TMS measures of cortical excitability and inhibition. Findings suggest that younger children have higher MTs which decrease with age and that this is more pronounced in depressed youth. These findings also suggest that LICI inhibition may increase with age and, like MTs, this is more pronounced in depressed youth. Present CSP, SICI, and ICF findings are inconclusive and warrant further study.

## Disclosures

Paul E. Croarkin has received research grant support from the National Institute of Mental Healthy, Brain and Behavior Research Foundation, National Alliance for Research on Schizophrenia and Depression (NARSAD) Great Neck, New York; and Pfizer Inc., New York, NY. He has served as a site subprincipal or principal investigator (without additional compensation) for Eli Lilly and Co, Indianapolis, Indiana; Forest Laboratories, Inc., New York, NY; Merck and Co, Inc., Whitehouse Station, New Jersey; and Pfizer Inc.

Mustafa M. Husain has received research support from the National Institute of Mental Healthy, Stanley Medical Research Institute, Cyberonics, Inc., Pfizer, Inc., Neuronetics Inc., Magstim; has served as a consultant for AstraZeneca; served on speaker bureau for AstraZeneca, Bristol-Meyers-Squibb, and Abbott Laboratories.

Graham J. Emslie has received research support from the National Institute of Mental Health, Biobehavioral Diagnostic Inc., BioMarin, Duke University, Eli Lilly, Forest Laboratories, GlaxoSmithKline, Mylan, and Somerset; has served as a consultant for Alkermes, Inc., Allergan, NCS Pearson (previously BioBehavioral Diagnostics Inc.), Bristol-Myers Squibb, Eli Lilly, Forest Laboratories, GlaxoSmithKline, INC Research Inc., Lundbeck, Merck, Pfizer, Seaside Therapeutics, Shire, the Texas Department of State Health Services, University of Miami, Valeant, and Wyeth; and was on the Speakers Bureau for Forest Laboratories.

In the last 5 years, Zafiris J. Daskalakis received research and equipment in-kind support for an investigator-initiated study through Brainsway Inc. and a travel allowance through Merck. Zafiris J. Daskalakis has also received speaker funding through Sepracor Inc., AstraZeneca and served on the advisory board for Hoffmann-La Roche Limited and Merck and received speaker support from Eli Lilly.

Drs. Paul A. Nakonezny, Charles P. Lewis, Michael J. Zaccariello, John E. Huxsahl, and Betsy D. Kennard have no disclosures.

### Conflict of interest statement

The authors declare that the research was conducted in the absence of any commercial or financial relationships that could be construed as a potential conflict of interest.
