# Supplementary material for: Developmental aspects of cortical excitability and inhibition in depressed and healthy youth: an exploratory study
Source: Front Hum Neurosci. 2014 Sep 2;8:669. doi: 10.3389/fnhum.2014.00669 (PMC4151107; doi:10.3389/fnhum.2014.00669)
Supplement: Supplementary file 1 [file DataSheet1.DOCX]

***Supplementary Material***

**Developmental aspects of cortical excitability and inhibition in depressed and healthy youth: An exploratory study**

Paul E. Croarkin^1*^, Paul A. Nakonezny^2,3^, Charles P. Lewis^1^, Michael J. Zaccariello^1^, John E. Huxsahl^1^, Mustafa M. Husain^4^, MD; Betsy D. Kennard^3^, Graham J. Emslie^3^,

Zafiris J. Daskalakis^5^

*^1^ Department of Psychiatry and Psychology, Mayo Clinic, Rochester, MN, USA*

*^2^Department of Clinical Sciences Division of Biostatistics, UT Southwestern Medical Center, Dallas, TX, USA*

*^3^Department of Psychiatry, UT Southwestern Medical Center, Dallas, TX, USA*

*^4^Department of Psychiatry and Behavioral Sciences, Duke University School of Medicine, Durham, NC, USA*

*^5^Department of Psychiatry, University of Toronto, Centre for Addiction and Mental Health, Toronto, Ontario, Canada*

**Corresponding Author:**

Paul E. Croarkin, DO, MSCS

Department of Psychiatry and Psychology

Mayo Clinic

200 First Street S.W.

Rochester, MN 55905, USA

1. **Supplementary Figures and Tables**

**Table 1. Relationship between age and TMS measures of cortical excitability and inhibition in all participants (N=46)**

| **Cortical Excitability or Inhibition Measure** | **r_s_ β** | **r_s_ p-value β p-value**  **(FDR) (FDR)** |
| --- | --- | --- |
| **Right Hemisphere MT** | **-0.4976 -0.0853** | **0.0007 0.0001**  **(0.0070) (0.0010)** |
| **Left Hemisphere MT** | **-0.4209 -0.0567** | **0.0044 0.0007**  **(0.0293) (0.0047)** |
| **Right Hemisphere CSP** | **0.0710 0.0084** | **0.6590 0.6354**  **(0.7753) (0.8519)** |
| **Left Hemisphere CSP** | **-0.0616 -0.0074** | **0.6980 0.6852**  **(0.7756) (0.8519)** |
| **Right Hemisphere SICI-2** | **-0.2361 -0.0781** | **0.1478 0.2323**  **(0.4389) (0.5808)** |
| **Left Hemisphere SICI-2** | **-0.1806 -0.0415** | **0.2465 0.3871**  **(0.5478) (0.7750)** |
| **Right Hemisphere SICI-4** | **-0.1161 -0.0207** | **0.4816 0.7296**  **(0.6020) (0.8519)** |
| **Left Hemisphere SICI-4** | **-0.1273 -0.0230** | **0.4158 0.6349**  **(0.6020) (0.8519)** |
| **Right Hemisphere ICF-10** | **0.1172 0.0101** | **0.4771 0.8093**  **(0.6020) (0.8519)** |
| **Left Hemisphere ICF-10** | **-0.0116 -0.0093** | **0.9411 0.7753**  **(0.9411) (0.8519)** |
| **Right Hemisphere ICF-15** | **0.1512 0.0227** | **0.3581 0.6256**  **(0.6020) (0.8519)** |
| **Left Hemisphere ICF-15** | **-0.1203 -0.0410** | **0.4419 0.1928**  **(0.6020) (0.5654)** |
| **Right Hemisphere ICF-20** | **0.2213 0.0894** | **0.1758 0.1731**  **(0.4395) (0.5654)** |
| **Left Hemisphere ICF-20** | **0.0276 0.0027** | **0.8613 0.9481**  **(0.9066) (0.9481)** |

*Abbreviations: MT, motor threshold; CSP, cortical silent period; SICI-2, short-interval intracortical inhibition with 2-millisecond interstimulus interval; SICI-4, short-interval intracortical inhibition with 4-millisecond interstimulus interval; ICF-10, intracortical facilitation with 10-millisecond interstimulus interval; ICF-15, intracortical facilitation with 15-millisecond interstimulus interval; ICF-20, intracortical facilitation with twenty second interstimulus interval.* Note. Cortical Excitability or Inhibition was Log Transformed.  Spearman correlation coefficient= r_s_; the unstandardized robust linear regression coefficient= β; p-value = two-tailed p-value unadjusted for multiple testing. FDR=False Discovery Rate.

**Table 2. Relationship between age and TMS measures of cortical excitability and inhibition in depressed youth (n=24).**

| **Cortical Excitability or Inhibition Measure** | **r_s_ β** | **r_s_ p-value β p-value**  **(FDR) (FDR)** |
| --- | --- | --- |
| **Right Hemisphere MT** | **-0.7054 -0.0925** | **0.0002 0.0001**  **(0.0020) (0.0010)** |
| **Left Hemisphere MT** | **-0.5468 -0.0576** | **0.0069 0.0035**  **(0.0345) (0.0175)** |
| **Right Hemisphere CSP** | **0.1877 0.0317** | **0.4279 0.1930**  **(0.5349) (0.3845)** |
| **Left Hemisphere CSP** | **-0.0964 -0.0002** | **0.6774 0.9948**  **(0.7969) (0.9948)** |
| **Right Hemisphere SICI-2** | **-0.1947 -0.0461** | **0.4107 0.6028**  **(0.5349) (0.7535)** |
| **Left Hemisphere SICI-2** | **-0.3054 -0.0544** | **0.1565 0.3779**  **(0.2845) (0.5760)** |
| **Right Hemisphere SICI-4** | **-0.0339 -0.0011** | **0.8871 0.9822**  **(0.9338) (0.9948)** |
| **Left Hemisphere SICI-4** | **-0.2787 -0.0542** | **0.1977 0.4320**  **(0.3295) (0.5760)** |
| **Right Hemisphere ICF-10** | **0.3297 0.0858** | **0.1557 0.1774**  **(0.2845) (0.3845)** |
| **Left Hemisphere ICF-10** | **0.0561 0.0191** | **0.7993 0.6978**  **(0.8881) (0.8209)** |
| **Right Hemisphere ICF-15** | **0.3891 0.0926** | **0.0900 0.2115**  **(0.2076) (0.3845)** |
| **Left Hemisphere ICF-15** | **-0.1846 -0.0355** | **0.3990 0.2620**  **(0.5349) (0.4367)** |
| **Right Hemisphere ICF-20** | **0.3974 0.1711** | **0.0827 0.0680**  **(0.2076) (0.2267)** |
| **Left Hemisphere ICF-20** | **0.0117 0.0119** | **0.9575 0.8366**  **(0.9575) (0.9296)** |

*Abbreviations: MT, motor threshold; CSP, cortical silent period; SICI-2, short-interval intracortical inhibition with 2-millisecond interstimulus interval; SICI-4, short-interval intracortical inhibition with 4-millisecond interstimulus interval; ICF-10, intracortical facilitation with 10-millisecond interstimulus interval; ICF-15, intracortical facilitation with 15-millisecond interstimulus interval; ICF-20, intracortical facilitation with twenty second interstimulus interval.* Note. Cortical Excitability or Inhibition was Log Transformed.  Spearman correlation coefficient= r_s_; the unstandardized robust linear regression coefficient= β; p-value = two-tailed p-value unadjusted for multiple testing. FDR=False Discovery Rate.

**Table 3. Relationship between age and TMS measures of cortical excitability and inhibition in healthy controls (n=22)**

| **Cortical Excitability or Inhibition Measure** | **r_s_ β** | **r_s_ p-value β p-value**  **(FDR) (FDR)** |
| --- | --- | --- |
| **Right Hemisphere MT** | **-0.3045 -0.0584** | **0.1795 0.0658**  **(0.9059) (0.6580)** |
| **Left Hemisphere MT** | **-0.2571 -0.0383** | **0.2604 0.1817**  **(0.9059) (0.7268)** |
| **Right Hemisphere CSP** | **0.0442 0.0055** | **0.8490 0.8379**  **(0.9612) (0.9622)** |
| **Left Hemisphere CSP** | **-0.0522 -0.0101** | **0.8222 0.6713**  **(0.9612) (0.9622)** |
| **Right Hemisphere SICI-2** | **-0.2289 -0.1089** | **0.3458 0.2785**  **(0.9059) (0.7503)** |
| **Left Hemisphere SICI-2** | **0.0188 -0.0097** | **0.9372 0.8924**  **(0.9612) (0.9622)** |
| **Right Hemisphere SICI-4** | **-0.2114 -0.0337** | **0.3850 0.7637**  **(0.9059) (0.9622)** |
| **Left Hemisphere SICI-4** | **0.0698 0.0342** | **0.7699 0.6055**  **(0.9612) (0.9622)** |
| **Right Hemisphere ICF-10** | **-0.1666 -0.0575** | **0.4954 0.3001**  **(0.9612) (0.7503)** |
| **Left Hemisphere ICF-10** | **-0.1452 -0.0549** | **0.5413 0.1659**  **(0.9612) (0.7268)** |
| **Right Hemisphere ICF-15** | **-0.2565 -0.0575** | **0.2891 0.2634**  **(0.9059) (0.7503)** |
| **Left Hemisphere ICF-15** | **-0.0428 -0.0560** | **0.8576 0.3503**  **(0.9612) (0.7784)** |
| **Right Hemisphere ICF-20** | **0.0771 0.0108** | **0.7538 0.9044**  **(0.9612) (0.9622)** |
| **Left Hemisphere ICF-20** | **0.1053 0.0064** | **0.6585 0.9141**  **(0.9612) (0.9622)** |

*Abbreviations: MT, motor threshold; CSP, cortical silent period; SICI-2, short-interval intracortical inhibition with 2-millisecond interstimulus interval; SICI-4, short-interval intracortical inhibition with 4-millisecond interstimulus interval; ICF-10, intracortical facilitation with 10-millisecond interstimulus interval; ICF-15, intracortical facilitation with 15-millisecond interstimulus interval; ICF-20, intracortical facilitation with twenty second interstimulus interval.* Note. Cortical Excitability or Inhibition was Log Transformed.  Spearman correlation coefficient= r_s_; the unstandardized robust linear regression coefficient= β; p-value = two-tailed p-value unadjusted for multiple testing. FDR=False Discovery Rate.

**Table 4. Relationship between age and TMS measures of LICI in all participants (n=33).**

| **Cortical Excitability or Inhibition Measure** | **r_s_ β** | **r_s_ p-value β p-value**  **(FDR) (FDR)** |
| --- | --- | --- |
| **Right Hemisphere LICI-100** | **-0.1872 -0.0491** | **0.3700 0.7097**  **(0.6020) (0.8519)** |
| **Left Hemisphere LICI-100** | **-0.1410 -0.0405** | **0.4493 0.7773**  **(0.6020) (0.8519)** |
| **Right Hemisphere LICI-150** | **-0.3236 -0.1482** | **0.1145 0.3875**  **(0.4389) (0.7750)** |
| **Left Hemisphere LICI-150** | **-0.2671 -0.1887** | **0.1536 0.1979**  **(0.4389) (0.5654)** |
| **Right Hemisphere LICI-200** | **-0.4883 -0.2450** | **0.0133 0.0014**  **(0.0565) (0.0070)** |
| **Left Hemisphere LICI-200** | **-0.6421 -0.2354** | **0.0001 0.0001**  **(0.0020) (0.0010)** |

*Abbreviations: LICI-100, Long-interval intracortical inhibition with 100-millisecond interstimulus interval; LICI-150, Long-interval intracortical inhibition with 150-millisecond interstimulus interval; LICI-200, Long-interval intracortical inhibition with 200-millisecond interval.* Note. Cortical Excitability or Inhibition was Log Transformed.  Spearman correlation coefficient= r_s_; the unstandardized robust linear regression coefficient= β; p-value = two-tailed p-value unadjusted for multiple testing. n=33, because 13 youth did not complete LICI testing. FDR=False Discovery Rate.

**Table 5. Relationship between age and TMS measures of LICI in depressed youth (n=14).**

| **Cortical Excitability or Inhibition Measure** | **r_s_ β** | **r_s_ p-value β p-value**  **(FDR) (FDR)** |
| --- | --- | --- |
| **Right Hemisphere LICI-100** | **-0.5302 -0.2186** | **0.0934 0.1739**  **(0.2076) (0.3845)** |
| **Left Hemisphere LICI-100** | **-0.5876 -0.3440** | **0.0573 0.0833**  **(0.2076) (0.2380)** |
| **Right Hemisphere LICI-150** | **-0.3791 -0.1675** | **0.2502 0.4092**  **(0.3849) (0.5760)** |
| **Left Hemisphere LICI-150** | **-0.5246 -0.4121** | **0.0799 0.0601**  **(0.2076) (0.2267)** |
| **Right Hemisphere LICI-200** | **-0.7664 -0.3539** | **0.0059 0.0007**  **(0.0345) (0.0047)** |
| **Left Hemisphere LICI-200** | **-0.9295 -0.2570** | **0.0001 0.0001**  **(0.0020) (0.0010)** |

*Abbreviations: LICI-100, Long-interval intracortical inhibition with 100-millisecond interstimulus interval; LICI-150, Long-interval intracortical inhibition with 150-millisecond interstimulus interval; LICI-200, Long-interval intracortical inhibition with 200-millisecond interval.* Note. Cortical Excitability or Inhibition was Log Transformed.  Spearman correlation coefficient= r_s_; the unstandardized robust linear regression coefficient= β; p-value = two-tailed p-value unadjusted for multiple testing. n=14, because 10 depressed youth did not complete LICI testing. FDR=False Discovery Rate.

**Table 6. Relationship between age and TMS measures of LICI in healthy controls (n=19).**

| **Cortical Excitability or Inhibition Measure** | **r_s_ β** | **r_s_ p-value β p-value**  **(FDR) (FDR)** |
| --- | --- | --- |
| **Right Hemisphere LICI-100** | **0.0511 0.1039** | **0.8621 0.5491**  **(0.9612) (0.9612)** |
| **Left Hemisphere LICI-100** | **0.1816 0.1382** | **0.4707 0.4358**  **(0.9612) (0.8716)** |
| **Right Hemisphere LICI-150** | **-0.3362 -0.0988** | **0.2398 0.7024**  **(0.9059) (0.9612)** |
| **Left Hemisphere LICI-150** | **-0.1168 -0.0008** | **0.6442 0.9965**  **(0.9612) (0.9965)** |
| **Right Hemisphere LICI-200** | **-0.2608 -0.1582** | **0.3678 0.1584**  **(0.9059) (0.7268)** |
| **Left Hemisphere LICI-200** | **-0.4527 -0.1958** | **0.0592 0.0239**  **(0.8773) (0.4780)** |

*Abbreviations: LICI-100, Long-interval intracortical inhibition with 100-millisecond interstimulus interval; LICI-150, Long-interval intracortical inhibition with 150-millisecond interstimulus interval; LICI-200, Long-interval intracortical inhibition with 200-millisecond interval.* Note. Cortical Excitability or Inhibition was Log Transformed.  Spearman correlation coefficient= r_s_; the unstandardized robust linear regression coefficient= β; p-value = two-tailed p-value unadjusted for multiple testing. n=19, because 3 healthy controls did not complete LICI testing. FDR=False Discovery Rate.
